# New Method of Penetrating the Antrum of Highmore

**Published:** 1893-09

**Authors:** 


### New Method of Penetrating the Antrum of Highmore.

Moure (*Gaz. Hop. de Toulouse, Epitome*) claims to overcome the objections of all the other methods by the following procedure: An area of the pitutiary membrane beneath the inferior turbinated bone is cleansed and anæsthetized with cocaine, and then by means of a galvano-cautery tip placed against it, the current is passed interrupted. The tip quickly penetrates the antrum.

After operation a small canula is placed in the opening. This is easily done, as the opening can be seen, owing to the retractile effect of the cocaine on the tissues. There is no loss of blood and no fear on the part of the patient. Moure prefers a sublimate solution 1 : 5000 for lavage of the cavity.

He acknowledges, however, that in some cases penetration by this method is difficult—even impossible. This may be owing to the thickness of the bony wall or to the existence of a bony column at the site of operation.

In these cases all that the cautery can do is to remove the mucous membrane and so prepare the way for the application of the trochar.

The caution is added that puncture by any method should be left as a last resort, for this procedure, if undertaken in patients with a purulent rhinitis, will often set up an empyema of the antrum, and that too in spite of all antiseptic precautions.

